# Cooccurrence of Chorea-Acanthocytosis and Mesial Temporal Sclerosis: A Possible Role of Caudate Nucleus

**DOI:** 10.1155/2017/2810925

**Published:** 2017-08-13

**Authors:** Mehri Salari, Alexander C. Lehn, Masoud Etemadifar, Seyed Amir Hejazi

**Affiliations:** ^1^Department of Neurology, School of Medicine, Qom University of Medical Science, Qom, Iran; ^2^Department of Neurology, Princess Alexandra Hospital, Brisbane, QLD, Australia; ^3^School of Medicine, University of Queensland, Brisbane, QLD, Australia; ^4^Department of Neurology, School of Medicine, Isfahan University of Medical Science, Isfahan, Iran

## Abstract

Chorea-acanthocytosis (ChAc) is an orphan disease, caused by mutations on chromosome 9. Epileptic seizures of mesial temporal origin can be a predominant symptom. We report on a 29-year-old woman with ChAc and bilateral MTS. Previously, few patients with coexisting ChAc and MTS were reported. The underlying pathophysiology is unknown, and further studies are needed.

## 1. Introduction

Chorea-acanthocytosis (ChAc) is caused by mutations in the VPS13A gene. This gene is located on chromosome 9 and codes for chorein. Approximately 40% of patients also suffer from epilepsy [1], and epileptic seizures of mesial temporal origin can be a predominant symptom in ChAc [2]. Only a few patients with mesial temporal lobe sclerosis (MTS) in ChAc have been reported in literature.

## 2. Case Report

We report on a 29-year-old woman who was referred to our movement disorders clinic. She also presented with obsessive-compulsive behavior and anxiety symptoms. Two years priorly, she had developed epilepsy, which was controlled with a combination of carbamazepine and divalproex sodium; no other medications had been used in the past. On examination she had orofacial dystonia with dystonic tongue protrusion interfering with her ability to eat. The patient exhibited intermittent tongue biting as well as vocal and motor tics. There was stocking-distribution sensory loss with depressed deep tendon reflexes. The patient showed several signs of previous self-mutilation attempts, including abrasions in the sole of the feet. There were not any Kayser-Fleischer ring and optic disk abnormality.

Brain MRI showed bilateral caudate atrophy and bilateral mesial temporal sclerosis. Serum as well as urine ceruloplasmin and copper levels were normal; creatine phosphokinase slightly increased (less than 500). Peripheral blood smear showed more than 5% of peripheral blood erythrocytes. The EEG showed bilateral spike-wave discharges, maximally in the temporal regions.

## 3. Discussion

To the best of our knowledge, a total of six patients suffering with chorea-acanthocytosis with MTS have been reported (Table 1). There are three reported cases with bilateral MTS, and we are reporting the fourth case. This association could just be incidental but ChAc is a rare condition with fewer than 5000 cases reported worldwide [3]. On the other hand, MTS might be a consequence of prolonged seizures in these patients. However Al-Asmi et al. suggested that familial temporal lobe epilepsy (TLE) could be a presenting feature of ChAc [4]. Our case and other previously reported cases support the hypothesis that the underlying genetic abnormality in ChAc might be a contributing factor to TLE and MTS [5]. Furthermore, animal model has demonstrated that VPS13A is expressed in hippocampus [6]. Another possible, yet unproven, explanation is dying back phenomenon involving corticostriatal pathway.

In their study from 2011 Riley et al. found that in TLE the caudate nucleus volume on the side of seizure onset is reduced compared to the other side. They concluded that frontostriatal impairment caused by caudate atrophy could lead to impaired executive functions in TLE patients [7]. Other disorders that cause caudate atrophy such as Huntington's disease also have an increased incidence of TLE. In these conditions caudate nucleus abnormalities may be conduit among dyskinesias, mesial temporal lobe epilepsy, and neuropsychiatric manifestations. Also anatomical connections between the amygdala and caudate nucleus support this theory. Additionally in 1962, researchers showed that stimulation of caudate nucleus can cause seizures [8].

Scheid et al. in 2009 described three cases of chorea-acanthocytosis. All patients had MTS on imaging and, notably, in two cases TLE was the sole clinical presentation of the disease [1], highlighting that temporal lobe seizure may be the presenting sign of ChAc.

In conclusion, we hypothesize that caudate dysfunction may be the culprit behind the development of TLE and hippocampal sclerosis. Further studies will be needed to confirm this theory.

## Figures and Tables

**Table 1:** Published cases with chorea-acanthocytosis and MTS.

Author	Year	MTS	Clinical Feature	Genetic or Western blot	Caudate atrophy
Al-Asmi et al. [4]	2005	Unilateral	Abnormal orofacial movement, CPS, neuropathy, behavioral and memory problems	Deletion of exons 70–73 of VPS13A, extending to exons 6-7 of GNA14	+

Scheid et al (3 cases) [1]	2009	Unilateral	GTC	Decrease chorein in Western blot	+
		Unilateral	Orofacial tic, CPS	Decrease chorein in Western blot	+
		Bilateral	CPS	Decrease chorein in Western blot	−

Bader et al. [2]	2011	Bilateral	Chorea, tic, orofacial dyskinesia, GTC, CPS	−	+

Mente et al. [6]	2017	Bilateral	Chorea, tic, orofacial dystonia, neuropathy, inattention, anxiety, CPS	Mutations in VPS13A	+

Current case	2017	Bilateral	Chorea, tic, orofacial dystonia, self-mutilation, GTC, anxiety, OCD, neuropathy	−	+
